# Analysis of nitric oxide synthase gene polymorphisms in neonatal respiratory distress syndrome among the Chinese Han population

**DOI:** 10.1186/1824-7288-40-27

**Published:** 2014-03-06

**Authors:** Wei Shen, Jiang Du, Bin Wang, Qiyi Zeng

**Affiliations:** 1 Center of pediatrics, Zhujiang Hospital, Southern Medical university, Guangzhou, Guangdong 510282, China

**Keywords:** Nitric oxide synthase, Respiratory distress syndrome, Preterm infants, Multiplex ligation detection reaction, Polymorphism

## Abstract

**Aim:**

To evaluate the association of NOS1 and NOS3 gene polymorphisms with the risk/severity of neonatal respiratory distress syndrome (RDS) among preterm infants.

**Methods:**

The patient group was 189 preterm infants diagnosed with RDS. The control group was 227 preterm neonates who did not develop RDS. NOS genotyping was performed using an improved multiplex ligation detection reaction (iMLDR) technique based on LDR.

**Results:**

It was found that genotype and allele frequencies of rs2682826 of the NOS1 gene and rs1799983 of the NOS3 gene were not significantly different between the RDS group and the control group. However, when the preterm infants were divided into two and three groups based on gestational age and birth weight, a study of the SNP rs1799983 of the NOS3 gene showed that the GG genotype and G allele frequencies were significantly increased in the RDS groups, the GT genotype and A allele were less frequent among the RDS groups in 26–32.9 weeks of gestational age and in a birth weight subgroup of <1.5 Kg.

**Conclusion:**

Our study raises the possibility that a genetic variation of NOS3 could be implicated in the pathophysiology of RDS in the Chinese Han population, especially in very preterm and very low birth weight infants.

## Introduction

Nitric oxide (NO) is a modulator of apoptotic and inflammatory cascades and endothelial permeability; it is synthesised by nitric oxide synthase (NOS) from L-arginine. In humans, neuronal NOS (nNOS) encoded by NOS1, inducible NOS (iNOS) encoded by NOS2A, and endothelial NOS (eNOS) encoded by NOS3 are three isoforms of NOS that are expressed in airway epithelium [1]. It has been identified that endogenous NO is vital for the decrease in pulmonary vascular resistance and the improvement in ventilation-perfusion matching after birth, which enables pulmonary hypertension to be reversed. NOS3 deficiency in mice causes abnormalities in lung morphogenesis, which results in respiratory distress and death within the first few hours of life [2]. Furthermore, clinical trials proved that inhaled nitric oxide had beneficial effects on respiratory outcomes [3,4].

NOS1 is located on chromosome 12q24.2 and is composed of 29 exons and 28 introns, which encompass more than 160 kb of genomic DNA [5]. The rs2682826 SNP is located in the 3′-UTR of exon 29 of the NOS1 gene. Given the known function of 3′-UTR in the degenerative stability and translational efficiency of mRNA, we cannot exclude the possibility that the rs2682826 SNP can be functionally important [6]. NOS3 is located on chromosome 7q35-36, it shows a single nucleotide polymorphism in exon 7, Glu298Asp (G894T, rs1799983), which results in a substitution of glutamic acid by aspartic acid at amino acid position 298 [7]. The Glu298Asp polymorphism was suggested to be associated with altered NOS3 enzyme activity, reduced NO production, and blunted endothelial-dependent vasodilation.

The study of genetic polymorphisms of NOS can help to understand individual variability in their susceptibility to the pathologies of pulmonary disease, especially RDS, which is the most frequent form of respiratory failure in preterm infants. There has been no study that investigated the role of NOS1 polymorphisms in RDS in preterm neonates. The association between the NOS3 gene and NRDS has been investigated in recent studies among Turkish population, but the data remain inconsistent [8,9] and the association between them among Chinese population is still unclear.

The Chinese population has over 1,30 billion people, in which the Han population is over 1,17 billion. Due to the variable genetic polymorphisms and distinct geographic residency and environment, predisposition and susceptibility to diseases vary. The aim of the present study is to determine whether the polymorphisms of NOS1 and NOS3 genes in preterm babies were associated with RDS in a Chinese Han population.

## Materials and methods

### Study population

A total of 416 patients were recruited from Zhujiang Hospital and Guangzhou Woman and Children’s Medical Center from October 2009 to February 2013 for the study. The RDS group was composed of 189 neonates from unrelated families. We defined RDS as the need for supplemental oxygen, a chest radiograph consistent with RDS and the need for continuous positive airway pressure or mechanical ventilation within the first 24 hours of life. The control group was composed of 227 preterm infants without RDS. Exclusion criteria were congenital anomalies, severe infection, and inherited metabolic disorders. Clinical data concerning gestational age, sex, birth weight, and maternal and neonatal clinical histories were obtained from the medical records. The gestational age of the infants were determined based on early ultrasound estimates of gestation and date of LMP. Informed consent was obtained from the parents of the infants, and the study protocol was approved by the local Ethics Committee of Southern Medical University.

### DNA isolation

Genomic DNA was extracted from 2 ml of EDTA-anticoagulated peripheral blood samples, using the QIAamp DNA Kit (Qiagen, Hilden, Germany ) according to the manufacturer’s instructions.

### SNP genotyping using the iMLDR™ technique

The SNP genotyping work was performed using an improved multiplex ligation detection reaction (iMLDR) technique, which was newly developed by Genesky Biotechnologies Inc. (Shanghai, China). This method was based on LDR. In this study, we applied the iMLDR technique on the genotyping of 2 SNP loci in one ligation reaction, which is the rs2682826 polymorphism of the NOS1 gene and the rs1799983 polymorphism of the NOS3 gene. A multiplex of PCR reactions was designed to amplify the 2 SNP loci. The first PCR reaction in 20 μl contained 1x PCR buffer (Takara), 3.0 mM Mg2+, 0.3 mM dNTP, 1 U of Hot-Start Taq DNA polymerase (Takara),1 μl of primer mixture 1 and ~20 ng of genomic DNA. The second PCR reaction in a 20 μl volume contained 1× GC Buffer I (Takara), 3.0 mM Mg2+, 0.3 mM dNTP, 1 U of Hot-Start Taq DNA polymerase (Takara),1 μl of primer mixture 2 and ~20 ng of genomic DNA. The primer information in two mixtures is described as follows.

rs1799983F:CAATGAGGGACCCTGGAGATGA,

rs1799983R:CCCAGTCAATCCCTTTGGTGCT;

rs2682826F:CAGGGGAACCCCAGAGAAAAAA;

rs2682826R:GCCTCCTGTCCTTGTCGCTGT.

The PCR program for both reactions was 95°C 2 min; 11 cycles × (94°C 20 s, 65°C-0.5°C/cycle 40 s, 72°C 1 min 30 s); 24 cycles × (94°C 20 s, 59°C 30 s, 72°C 1 min 30 s); and 72°C 2 min; hold at 4°C. The two PCR products were equally mixed and purified by 1 U of shrimp alkaline phosphatase’s digestion at 37°C for 1 hr and at 75°C for 15 min. The ligation reaction in 20 μl contains 1 × ligation buffer, 80 U of Taq DNA Ligase (NEB), 1 μl of labeling oligo mixture, 2 μl of probe mixture and 5 μl of purified PCR product mixture. The oligo or probe information in these two mixtures is described as follows:

rs1799983FG:TTCCGCGTTCGGACTGATATTGCTGCAGGCCCCAGATCAG,

rs1799983FP:CCCCCAGAACTCTTCCTTCTGCTTTTTTTTTTT,

rs1799983FT:TACGGTTATTCGGGCTCCTGTTGCTGCAGGCCCCAGATCAT;

rs2682826RA:TACGGTTATTCGGGCTCCTGTTGCCGACAAGGGCAACTGAT,

rs2682826RG:TTCCGCGTTCGGACTGATATTGCCGACAAGGGCAACTGAC,

rs2682826RP:GGGTGCATGAAACCACTGGATTTTTTTTTT.

The ligation cycling program was 95°C 2 min; 38 cycles × (94°C 1 min, 56°C 4 min); hold at 4°C. A 0.5 μl of ligation product was loaded in ABI 3730 × l, and the raw data were analysed by GeneMapper 4.1. All of the primers, probes and labeling oligos were designed by and ordered from Genesky Biotechnologies Inc. (Shanghai, China).

### Statistical analysis

Statistical analysis was performed using the SPSS package program (version 15.0 Chicago, IL, 2006). Data were expressed as the mean ± SD or percentage. Allelic frequencies were calculated by the gene-counting method. A statistical comparison of two groups was performed by an unpaired Student’s t-test, while APGAR scores were compared with the Mann–Whitney U-test. A chi-square test was used to test the expected type frequencies, assuming Hardy-Weinberg equilibrium. The chi-square test or Fisher’s exact test was used for calculation of the significance of differences in genotype and allele frequencies. P < 0.05 was considered to be statistically significant.

## Results

The RDS group was composed of 189 preterm infants, and eight of the 189 infants (4.2%) died in the follow-up period due to RDS. The control group was composed of 227 preterm infants without RDS and five (2.2%) died for other diseases. All of the infants come from the Han population in south China. The genotypes in the population were in Hardy-Weinberg equilibrium (P > 0.05, data not shown). The demographic characteristics of the RDS and control groups are summarised in Table 1. Gestational age, birth weight, and the Apgar scores at the 5th minute were found to be significantly different from the controls (p < 0.001). There were no differences in gender, maternal age, mode of delivery, the rates of premature rupture of membranes (PROM), the use of antenatal steroid and supplementary surfactant (Table 1).

**Table 1:** Clinical and demographic characteristics of infants with and without RDS

	**Control group (n = 227)**	**RDS group (n = 189)**	**P value**
Gestational age (weeks)	35.1 ± 2.0	32.2 ± 2.5	0.000
Birth weight (g)	2388 ± 625	1813 ± 504	0.000
Gender (male/female)	123/104	118/71	0.090
Apgar score (5^th^ min)	8.1 ± 1.3	4.3 ± 1.2	0.000
Mode of delivery (V/CS)	108/119	76/113	0.132
Maternal age (years)	27.7 ± 5.4	29.0 ± 5.6	0.225
PROM >18 hours	71/156	63/126	0.655
Use of antenatal steroid	39/188	44/145	0.121
Use of surfactant	158/69	147/42	0.061

As shown in Tables 2 and 3, for both NOS1-rs2682826 and NOS3-1799983 SNP loci, there were no statistically significant differences in the genotype and allele frequencies between the control and RDS groups (P > 0.05). However, when the preterm neonates were divided into two groups by gestational age and birth weight, the result vary. The genotype and allele frequencies were not significantly different between the control and RDS groups in any subgroup of GA and BW for the NOS1-2682826 loci (p > 0.05) (Table 3).

**Table 2:** Genotypes and allele frequencies of the polymorphisms of the NOS genes in RDS and control groups and their association with the risk of RDS

**Genotypes/Alleles**	**Control group (n = 227) n (%)**	**RDS group (n = 189) n (%)**	**P value**	**OR (95% CI)**
rs2682826 (NOS1)				
GG	99 (43.6)	86 (45.5)		
GA	107 (47.1)	75 (39.7)	0.308	0.807 (0.534–1.200)
AA	21 (9.3)	28 (14.8)	0.201	1.535 (0.813–2.897)
G allele	305 (67.2)	247 (65.3)		
A allele	149 (32.8)	131 (34.7)	0.577	1.086 (0.814–1.449)
rs1799983 (NOS3)				
GG	134 (59.0)	127 (67.2)		
GT	88 (38.8)	57 (30.2)	0.070	0.683 (0.453–1.032)
TT	5 (2.2)	5 (2.6)	1.000	1.055 (0.298–3.731)
G allele	356 (78.4)	311 (82.3)		
T allele	98 (21.6)	67 (17.7)	0.164	0.783 (0.554–1.106)

**Table 3:** Genotype and allele frequencies of the rs2682826 polymorphism of the NOS1 gene in RDS and the control groups among different gestational ages and birth weights

**Genotype/Allele**	**Control group (n = 227)**	**RDS group (n = 189)**	**P value**	**OR (95% CI)**
Gestational age				
26w ≤ GA < 33 W	n = 107	n = 93		
GG	45 (42.1)	41 (44.1)		
GT	51 (47.7)	36 (38.7)	0.405	0.775 (0.425–1.413)
TT	11 (10.3)	16 (17.2)	0.294	1.596 (0.664–3.836)
G allele	141 (65.9)	118 (63.4)		
T allele	73 (34.1)	68 (36.6)	0.609	1.113 (0.738–1.679)
33w ≤ GA < 37 W	n = 120	n = 96		
GG	54 (45.0)	45 (46.9)		
GT	56 (46.7)	39 (40.6)	0.536	0.836 (0.473–1.476)
TT	10 (8.3)	12 (12.5)	0.440	1.440 (0.569–3.642)
G allele	164 (68.3)	129 (67.2)		
T allele	76 (31.7)	63 (32.8)	0.800	1.054 (0.702–1.581)
Birth weight				
BW < 1.5	n = 63	n = 44		
GG	25 (39.7)	21 (47.7)		
GT	33 (52.4)	19 (43.2)	0.360	0.685 (0.305–1.540)
TT	5 (7.9)	4 (9.1)	1.000	0.952 (0.226–4.008)
G allele	83 (65.9)	61 (69.3)		
T allele	43 (34.1)	27 (30.7)	0.597	0.854 (0.477–1.532)
1.5 < BW < 2.5	n = 86	n = 70		
GG	36 (41.9)	31 (44.3)		
GT	42 (48.8)	32 (45.7)	0.718	0.885 (0.455–1.720)
TT	8 (9.3)	7 (10.0)	0.978	1.106 (0.331–3.122)
G allele	114 (66.3)	94 (67.1)		
T allele	58 (33.7)	46 (32.9)	0.872	0.962 (0.599–1.545)
BW > 2.5	n = 85	n = 75		
GG	38 (44.7)	34 (45.3)		
GT	32 (37.6)	24 (32.0)	0.623	0.838 (0.415–1.693)
TT	15 (17.6)	17 (22.7)	0.578	1.267 (0.550–2.918)
G allele	108 (63.5)	92 (61.3)		
T allele	62 (36.5)	58 (38.7)	0.686	1.098 (0.698–1.728)

For the NOS3-1799983 SNP loci, in the 26–32.9 weeks of gestational age subgroup, GG and GT genotype frequencies were 41.3% and 56.3% of the control group and 65.8% and 30.8% of the RDS group, respectively (P = 0.001), there were marked increases in the G allele frequencies in the RDS groups in this gestational age group (P < 0.05). In the 33–36.9 weeks of gestational age subgroup, the difference in genotype distributions and allele frequencies did not reach statistical significance (p > 0.05) (Table 4). For the subgroups according to birth weight, GG and GT genotype frequencies were 47.6% and 52.4% of the control group and 68.8% and 23.4% of the RDS group in the birth weight <1.5 Kg subgroup, respectively (P < 0.05), whereas there were no significant differences in the genotype or allele distributions between the control and RDS groups in both the 1.5-2.5 Kg of birth weight and birth weight >2.5 subgroups (P > 0.05) (Table 4).

**Table 4:** Genotype and allele frequencies of the rs1799983 polymorphism of the NOS3 gene in RDS and the control groups among different gestational ages and birth weights

**Genotype/Allele**	**Control group (n = 227)**	**RDS group (n = 189)**	**P value**	**OR (95% CI)**
Gestational age				
26w ≤ GA < 33 W	n = 80	n = 117		
GG	33 (41.3)	77 (65.8)		
GT	45 (56.3)	36 (30.8)	0.001	0.343 (0.188-0.624)
TT	2 (2.4)	4 (3.4)	1.000	0.857 (0.150-4.911)
G allele	111 (69.4)	190 (81.2)		
T allele	49 (30.6)	44 (18.8)	0.007	0.525 (0.328-0.839)
33w ≤ GA < 37 W	n = 147	n = 72		
GG	101 (68.7)	50 (69.4)		
GT	43 (29.3)	21 (29.2)	1.000	0.987 (0.530-1.838)
TT	3 (2.0)	1 (1.4)	1.000	0.673 (0.068-6.639)
G allele	245 (83.3)	121 (84.0)		
T allele	49 (16.7)	23 (16.0)	0.892	0.950 (0.553-1.633)
Birth weight				
BW < 1.5	n = 21	n = 64		
GG	10 (47.6)	44 (68.8)		
GT	11 (52.4)	15 (23.4)	0.031	0.310 (0.110-0.875)
TT	0 (0)	5 (7.8)	-	-
G allele	31 (73.8)	103 (80.5)		
T allele	11 (26.2)	25 (19.5)	0.387	0.684 (0.303-1.545)
1.5 < BW < 2.5	n = 105	n = 106		
GG	73 (69.5)	71 (67.0)		
GT	30 (28.6)	35 (33.0)	0.554	1.200 (0.667-2.157)
TT	2 (1.9)	0 (0)	-	-
G allele	176 (83.8)	177 (83.5)		
T allele	34 (16.2)	35 (16.5)	1.000	1.024 (0.611-1.715)
BW > 2.5	n = 101	n = 19		
GG	51 (50.5)	12 (63.2)		
GT	47 (46.5)	7 (36.8)	0.455	0.633 (0.230-1.743)
TT	3 (3.0)	0 (0)	-	-
G allele	149 (73.8)	31 (81.6)		
T allele	53 (26.2)	7 (18.4)	0.414	0.635 (0.264-1.527)

We compared clinical features and the severity of illness in the groups of RDS infants with the rs1799983 (GG) and rs1799983 combined genotype (GT + TT), and we observed that the frequencies of the rs1799983 polymorphism did not markedly influence the complications, duration of oxygen therapy, mechanical ventilation (including CPAP) and hospitalisation. There were also no statistically significant differences between the demographic characteristics of the two groups except for gestational age, birth weight and use of antenatal steroid between the genotypes (Table 5).

**Table 5:** Effect of rs1799983 polymorphism of NOS3 gene on characteristics and severity of illness among RDS infants

	**GG genotype(n = 129 )**	**GT + TT genotype(n = 60 )**	**P value**
Gestational age (weeks)	33.0 ± 2.5	31.3 ± 2.1	0.000
Birth weight (g)	2050 ± 575	1753 ± 373	0.000
Gender (male/female)	79/50	39/21	0.619
Mode of delivery (V/CS)	47/82	29/31	0.120
PROM > 18 h	41/88	22/38	0.507
Use of antenatal steroid	24/105	20/40	0.026
Use of surfactant	98/31	49/11	0.380
Days of oxygen	38.3 ± 23.4	39.8 ± 25.7	0.135
Days of ventilation	20.1 ± 13.4	19.7 ± 15.6	0.407
Days of hospitalization	43.8 ± 21.6	42.2 ± 17.3	0.075
Complications			
PDA	27/102	18/42	0.173
BPD	32/97	11/49	0.323
ROP	11/118	9/51	0.178
IVH	14/115	2/58	0.098

V: Vaginal delivery; CS: cesarean section; PROM: premature rupture of membranes; PDA: patent ductus arteriosus; BPD: bronchopulmonary dysplasia; ROP: retinopathy of prematurity; IVH: intraventricular hemorrhage.

## Discussion

RDS is a multifactorial disease, and several genetic factors could interact with environmental factors in determining the risk of the disease. Both the incidence and severity of RDS have decreased markedly since the introduction of antenatal steroids and pulmonary surfactant replacement therapy, yet the incidence of complications remains a cause of concern [10]. Several mutations and polymorphisms within various genes, especially surfactant-associated genes, have previously been suggested to be modifiers of the risk for and course of neonatal RDS among preterm infants. Investigators have reported a higher risk for RDS in neonates who carry the surfactant protein A1(SP-A1) 6A2 allele [11], surfactant protein B(SP-B) 131Thr allele [12], or surfactant protein C(SP-C) 138Asn allele [13]. There is also an association between a synonymous code SNP rs323043 of the ATP-binding cassette transporter A3 gene (ABCA3) and the development of RDS [14]. On the other hand, the presence of inflammatory genes, including the MaNOS1e-binding lectin gene (MBL2), tumour necrosis factor (TNF) and the interferon gamma gene (IFNG), did not alter the risk of RDS [15]. Moreover, the 6A3 allele of surfactant protein A1(SP-A1) was less common among preterm infants with RDS and was absent among those with severe RDS, which suggests a possible protective role for this allele or other genes with which it is in linkage disequilibrium in RDS [11]. Glutathione-S-transferase-P1 (GSTP1) I105V polymorphism and Angiotensin converting enzyme (ACE) gene deletion/deletion polymorphism have also been reported to be protective factors for respiratory distress in preterm infants, and another study showed that angiotensin-converting enzyme gene polymorphism did not impact the risk or severity of persistent pulmonary hypertension of the newborn among infants >34 weeks GA [16-18]. Many of these associations with RDS have not been tested in multiple independent populations or have not been consistently replicated across studies.

Nitric oxide mediates multiple physiological functions, including neurotransmission, immunoregulation, angiogenesis, antiplatelet activity, and surfactant maturation or secretion. Given the many physiological roles for NO and its rapid reaction and inactivation in cellular systems, strict control of NO production is crucial for its selective actions. NO is critical for the maturation of pulmonary structure and function. Research in animals has suggested that NOS3 gene-deficient mice suffered defective lung vascular development and fatal respiratory distress [2].

Several polymorphic variations of the NOS3 gene are now known and have been investigated with respect to disease risk. A few literature reports have revealed that the NOS3 gene Glu298Asp (rs1799983) polymorphism is associated with an increased incidence of cardiovascular disorders and cerebrovascular diseases [19]. Godfrey V et al.’s study of the functional consequence of the Glu298Asp polymorphism in young healthy volunteers showed that this loci was associated with a blunted endothelial-dependent vasodilation, which possibly results from decreased NO synthesis [20]. Savvidou et al. showed that the Glu298Asp polymorphism was associated with differences in endothelium-dependent dilation at a 12-week gestation, thus implicating genetic factors in the normal vascular adaptation to pregnancy [21].

This study is the first to examine the impact of the NOS1 gene polymorphism on the risk and severity of RDS in preterm infants; we found no significant difference in the genotype or allele frequencies between the control and the RDS infants. Similarly, the difference in the genotype distributions of the NOS3 gene between the two groups did not reach statistical significance. However, when the preterm infants were divided into two groups by gestational age and three groups by birth weight, we found that, for the NOS3 gene, the GG genotype and G allele increased while the GT genotype and T allele decreased, and infants with the GG genotype and G allele of NOS3 were less likely to have development of RDS in the 26–32.9 weeks of gestational age subgroup and the birth weight <1.5 Kg subgroup.

Two meta-analyses showed that inhaled nitric oxide could alleviate persistent pulmonary hypertension in the neonates, but for the treatment of respiratory failure in preterm infants, routine use of inhaled nitric oxide cannot be recommended [22,23]. It is reported that common variants in the NO synthesis pathway genes jointly contribute to differences in exhaled nitric oxide (FeNO) levels in children, which is a biomarker of airway inflammation, most likely through differential gene expression [24-26]. In Gravesande et al.’ s study, the T allele is associated with lower NO levels, which demonstrates that the Glu298Asp polymorphism is functionally relevant and could be a reason for the low FeNO levels [27]. In placental tissue, Wang and coworkers showed that the Glu298Asp variant influences NOS3 expression and enzyme activity [28]. One possible mechanism by which the GG genotype and G allele of NOS3 could influence the risk of RDS in very preterm and very low birth weight infants in the present study could be through the generation of low levels of FeNO, which could mitigate lung inflammation and interfere with pulmonary vascular remodelling, thus determination of FeNO or plasma levels of NO metabolites is worthy for further study. This polymorphism leads to the alteration of NOS3 enzyme activity and is associated with the reduced basal NO product.

A limitation of our study is that our sample size was not large enough to compare the control and RDS groups, which could lead to false-negative results in association studies. The sample was not large enough to explore gene by gene interactions, it is possible that having more than one susceptibility gene could result in different respiratory outcomes. Furthermore, only two polymorphisms that are potentially involved in RDS pathogenesis have been considered in this paper; thus, the roles of other SNPs in NOS genes or in other different genes cannot be excluded.

In conclusion, our data suggest the lack of an association between the rs2682826 polymorphism of the NOS1 gene and the development of RDS in a Chinese Han population. We observed that there is a gestational age and birth weight -related association between the rs1799983 polymorphism of the NOS3 gene and the development of RDS in this population. However, further investigations of NOS3 genetic variations in combination with other relevant genes (especially those involved in pulmonary surfactant and Lipid metabolism) will most likely provide more mechanistic insight into RDS processes. Additional identification of how NOS3 genetic variation is translated to altered enzyme and/or cellular function will also be informative. This pilot study could offer data on which further studies can be planned. Very large-scale studies of genetic, environmental and obstetric factors will be needed before effective preventative strategies can be devised.

## Abbreviations

RDS: Respiratory distress syndrome; NOS: Nitric oxide synthase; iMLDR: Improved multiplex ligation detection reaction; FeNO: Fractional exhaled nitric oxide; SNP: Single nucleotide polymorphism.

## Competing interests

The authors declare that they have no competing interests.

## Authors’ contributions

WS carried out the molecular genetic studies, participated in the sequence alignment and drafted the manuscript. JD participated in the sequence alignment. BW participated in the design of the study and performed the statistical analysis. QZ conceived of the study, and participated in its design and coordination and helped to draft the manuscript. All authors read and approved the final manuscript.
